# Metabolomics-Driven Identification of the Rate-Limiting Steps in 1-Propanol Production

**DOI:** 10.3389/fmicb.2022.871624

**Published:** 2022-04-14

**Authors:** Toshiyuki Ohtake, Naoki Kawase, Sammy Pontrelli, Katsuaki Nitta, Walter A. Laviña, Claire R. Shen, Sastia P. Putri, James C. Liao, Eiichiro Fukusaki

**Affiliations:** ^1^Department of Biotechnology, Graduate School of Engineering, Osaka University, Suita, Japan; ^2^Department of Chemical and Biomolecular Engineering, University of California, Los Angeles, Los Angeles, CA, United States; ^3^Institute of Molecular Systems Biology, ETH Zürich, Zurich, Switzerland; ^4^Microbiology Division, Institute of Biological Sciences, University of the Philippines Los Baños, Los Baños, Philippines; ^5^Department of Chemical Engineering, National Tsing Hua University, Hsinchu, Taiwan; ^6^Industrial Biotechnology Initiative Division, Institute for Open and Transdisciplinary Research Initiatives, Osaka University, Suita, Japan; ^7^Osaka University Shimadzu Omics Innovation Research Laboratories, Osaka University, Suita, Japan

**Keywords:** metabolomics, rate-limiting steps, *Escherichia coli*, 1-propanol, 2-ketobutyrate, YqhD

## Abstract

The concerted effort for bioproduction of higher alcohols and other commodity chemicals has yielded a consortium of metabolic engineering techniques to identify targets to enhance performance of engineered microbial strains. Here, we demonstrate the use of metabolomics as a tool to systematically identify targets for improved production phenotypes in *Escherichia coli*. Gas chromatography/mass spectrometry (GC/MS) and ion-pair LC-MS/MS were performed to investigate metabolic perturbations in various 1-propanol producing strains. Two initial strains were compared that differ in the expression of the citramalate and threonine pathways, which hold a synergistic relationship to maximize production yields. While this results in increased productivity, no change in titer was observed when the threonine pathway was overexpressed beyond native levels. Metabolomics revealed accumulation of upstream byproducts, norvaline and 2-aminobutyrate, both of which are derived from 2-ketobutyrate (2KB). Eliminating the competing pathway by gene knockouts or improving flux through overexpression of glycolysis gene effectively increased the intracellular 2KB pool. However, the increase in 2KB intracellular concentration yielded decreased production titers, indicating toxicity caused by 2KB and an insufficient turnover rate of 2KB to 1-propanol. Optimization of alcohol dehydrogenase YqhD activity using an ribosome binding site (RBS) library improved 1-propanol titer (g/L) and yield (g/g of glucose) by 38 and 29% in 72 h compared to the base strain, respectively. This study demonstrates the use of metabolomics as a powerful tool to aid systematic strain improvement for metabolically engineered organisms.

## Introduction

Engineering microbes to have desired production phenotypes requires optimization of many physiological constraints (Pontrelli et al., 2018). These include the ability to express non-native enzymes, supply of required cofactors, precursor availability, oxygen tolerance, and accumulation of toxic intermediates or products. While rational design strategies prove useful in addressing many of these factors, there often remains a need for further optimization to improve production.

Metabolomics, a technology that involves the global quantitative assessment of metabolites in a biological system, can detect complex biological changes using statistical multivariate pattern recognition methods (chemometrics) (Putri et al., 2013). The system-wide analysis provided by metabolomics can be used to gain a deeper understanding of how various genetic modifications affect production phenotypes. Observations made with metabolomics have led to strain modifications that successfully increased biofuel production titers (Nitta et al., 2017; Ohtake et al., 2017; Fathima et al., 2018). For improvement of 1-butanol production in *E. coli*, a metabolomics-based approach was used to identify an imbalance of Coenzyme A caused by a *Clostridial* butanol production pathway. Further genetic modifications based on observations made using metabolomics allowed a production increase from 15 to 18.3 g/L of butanol (Ohtake et al., 2017). Similarly, an orthogonal partial least squares/projections to latent structures (PLS) model-based metabolomics approach was used to identify acetyl-CoA as the rate limiting step of 1-butanol production (Nitta et al., 2017).

**FIGURE 1:**
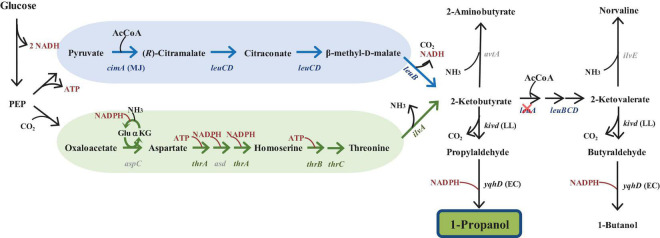
Illustration of the keto-acid pathway for 1-propanol synthesis. Schematic illustration of the threonine pathway (denoted by the green arrow) and the citramalate pathway (denoted by the blue arrow) for the synthesis of 1-propanol in *E. coli* from glucose as previously described in Shen and Liao (2013). Genes not overexpressed are noted in gray font. Cross mark indicates that the pathway was blocked via gene deletion. The deleted gene encode for 2-isopropylmalate synthase (*leuA*). PEP, phosphoenolpyruvate; cimA, citramalate synthase from *Methanococcus jannaschii* (MJ); KivD, alpha-ketoisovalerate decarboxylase from *Lactococcus lactis* (LL); YqhD, alcohol dehydrogenase from *Escherichia coli* (EC).

In this work, we employed a metabolomics-based approach to identify targets for improving production of 1-propanol in *E. coli*. 1-Propanol is recognized as an important compound that is industrially valuable for both energy applications and as a C_3_ chemical feedstock (Liao et al., 2016). Production of 1-propanol has been achieved via the keto acid pathway (Atsumi and Liao, 2008; Shen and Liao, 2013), which offers several advantages owing to its universality and compatibility with the host. 2-Keto acids are intermediates in amino acid biosynthesis pathways that can be found in almost all living organisms. The theoretical yield of 1-propanol was enhanced by combining the native threonine pathway with a heterologous citramalate pathway using glucose as a substrate (Shen and Liao, 2013). Different cofactor requirements are exhibited by each pathway individually. The threonine pathway requires 3 NAD(P)H and 2 ATP as inputs, while the citramalate pathway produces 1 NAD(P)H and 1 ATP as outputs (Figure 1). When the overall net reactions of each pathway are combined, the individual cofactor requirements can complement one another, resulting in a synergistic effect that improves the theoretical maximum yield. The efficacy of this synergistic approach has proven successful in improving the observed 1-propanol yield and productivity (Shen and Liao, 2013).

With this production strategy, each pathway converges into a common intermediate, 2-ketobutyrate (2KB), which is then converted into 1-propanol through reactions catalyzed by alpha-ketoisovalerate decarboxylase (KivD) and alcohol dehydrogenase (YqhD) (Figure 1). 2-Ketobutyrate is also a native precursor in the *E. coli* leucine biosynthesis pathway and subsequently converted into 2-ketovalerate. Because of the promiscuous nature of KivD and YqhD, 1-butanol is also formed as a side product. However, in this work, we focus solely on improving production of 1-propanol.

It has been observed that overexpression of the citramalate pathway in conjunction with the native threonine pathway exhibits an increased productivity (hereafter referred to as strain SYN3). Yet, there is no observed change in titer when the threonine pathway is further overexpressed over basal metabolic levels (hereafter called strain SYN12) (Shen and Liao, 2013). One recent study uses snapshot metabolomic profiling and dynamic metabolic turnover analysis to determine why the presence or overexpression of the citramalate or threonine pathways contributes to this change in productivity (Putri et al., 2018). The strains used in their study were SYN2 (citramalate pathway overexpressed and native threonine pathway deleted), SYN6 (threonine pathway overexpressed), and SYN12 (both pathways overexpressed). Notably, this study identified the importance of the PEP/pyruvate ratio, which affects glucose consumption, and how ammonia assimilation affects the rate of the threonine pathway. Extending these findings, we here build on this study by increasing 1-propanol titer in SYN12. First, derivatives of 1-propanol producing strains were cultivated under the optimal condition for production. Snapshot non-targeted metabolome analysis using gas chromatography/mass spectrometry (GC/MS) and widely targeted metabolome analysis using ion-pair LC/MS/MS were performed. The data sets obtained from GC/MS and ion-pair liquid chromatography (LC)/MS/MS (LC/MS/MS) were subjected to multivariate analyses to extract the most meaningful metabolites corresponding to the phenotype. Following the analysis of important metabolites, the corresponding metabolic pathways were examined, and gene targets were systematically selected to construct strains with improved production phenotypes.

## Materials and Methods

### Bacterial Strains

*Escherichia coli* strains, plasmids and primers used in this study are summarized in Table 1 and Supplementary Table 1. XL-1 Blue (Stratagene, La Jolla, CA, United States) was used to propagate all plasmids. Host gene deletion of *avtA* was achieved with P1 transduction using the Keio collection strains (Baba et al., 2006) as donor and strain CRS59 (Shen and Liao, 2013) as recipient. The kan*^R^* inserted into the target gene region was removed with pCP20 (Datsenko and Wanner, 2000) in between each consecutive knock out. Then, removal of the gene segment was verified by colony PCR using the appropriate primers.

**TABLE 1:** Strains and plasmids used in this work.

Name	Relevant characteristics	Source
***E. coli* strain**		
BW25113	*rrnB*_T14_ Δ*lacZ*_WJ16_ Δ*hsdR*514 Δ*araBAD*_AH33_ Δ*rhaBAD*_LD78_	Datsenko and Wanner, 2000
JCL16	BW25113/F’ [Δ*traD*36 *proAB*^+^ Δ*lacI*^q^ZΔM15 (Tet^r^)]	Atsumi et al., 2008
CRS59	JCL16 Δ*ilvB* Δ*ilvI* Δ*leuA*	Shen and Liao, 2013
CRS60	JCL16 Δ*ilvB* Δ*ilvI*Δ*leuA*Δ*avtA*	Shen and Liao, 2013
CRS-SYN3	CRS59/pSA138+pAFC52	Shen and Liao, 2013
CRS-SYN12	CRS59/pAFC66+pCS49+pAFC52	Shen and Liao, 2013
CRS-SYN14	CRS60/pAFC66+pCS49+pAFC52	Shen and Liao, 2013
CRS-SYN17	CRS59/pAFC66+pCS164+pAFC52	This study
CRS-SYN12-KivD34	CRS59/pKN2+pCS49+pAFC52	This study
CRS-SYN12-YqhD18	CRS59/pKN3+pCS49+pAFC52	This study
**Plasmid**		
pSA138	P_L_lacO_1_::*kivd* (LL)-*yqhD* (EC); ColE1 ori; Amp^R^	Atsumi and Liao, 2008
pAFC52	P_L_lacO_1_::*cimA2*Δ (MJ)-*leuBCD* (EC); p15A ori; Kan^R^	Shen and Liao, 2013
pAFC66	P_L_lacO_1_::*kivd* (LL)-*yqhD* (EC)-*ilvA* (CG); ColE1 ori; Amp^R^	Shen and Liao, 2013
pCS49	P_L_lacO_1_::*thrA^fbr^BC* (EC ATCC 21277); pSC101 ori; Spec^R^	Shen and Liao, 2008
pCS164	P_L_lacO_1_::*thrA^fbr^BC* (EC ATCC 21277)-*tpiA* (EC); pSC101 ori; Spec^R^	This study
pKN2	P_L_lacO_1_::*kivd* (LL)-*yqhD* (EC)-*ilvA* (CG); ColE1 ori; Amp^R^ (Improved kivd RBS strength)	This study
pKN3	P_L_lacO_1_::*kivd* (LL)-*yqhD* (EC)-*ilvA* (CG); ColE1 ori; Amp^R^ (Improved yqhD RBS strength)	This study
**YqhD RBS sequence**		
SYN12	GCATGCAGGAGAAAGGTCAC	This study
SYN12-YqhD18	GCCGCACCTCGCGAGTCAGAAAGGAGGAACCCGC	This study

### Plasmid Construction

Plasmids were constructed by assembling the three PCR fragments (Supplementary Table 1) similar to the previously described protocol (Ohtake et al., 2017).

### Ribosome Binding Site (RBS) Library Construction

pAFC66 was used as a template to construct a KivD and YqhD library with total eight variants. They were assembled by amplification of the following primers and assembly of the resulting PCR products (Supplementary Table 1). The KivD library was generated from a degenerate RBS library sequence to create eight variants, while the YqhD library was generated from three degenerate RBS library sequences that were pooled together in an equimolar ratio for eight total variants.

### Production Media and Cultivation Condition

Production of 1-propanol was conducted as described previously (Shen and Liao, 2013). For productions which lasted more than 2 days, 18 g/L of glucose was fed into the culture and pH of production culture was re-adjusted back to 7 using 10 M NaOH every day.

### Alcohol Dehydrogenase Enzyme Assay

Three independent cultures of SYN12 and SYN12-YqhD18 were grown and induced in production media as described above and harvested at 24 h by centrifugation. The pellets were then lysed by BugBuster (Millipore) and centrifuged at 13,200 rpm for 10 min at 4°C. The supernatant was collected and used as an input for an enzyme assay. The enzyme reaction contained 10 mM propionaldehyde and 1 mM NADPH in 100 mM PBS (pH6.4). The assay was initiated with addition of cell lysate and OD_340_ was measured over time.

### Sample Collection and Sample Preparation for Extracellular and Intracellular Metabolites Analysis

Samples were taken by opening the screw caps of the flasks, and culture broths were centrifuged at 4°C for 5 min, 14,000 rpm to retrieve the supernatant for extracellular metabolite detection. For intracellular metabolome analysis, five OD_600_ units of cells were collected by fast filtration using a 0.45 μm pore size, 47 mm diameter nylon membrane (Millipore). The cells were immersed in liquid nitrogen immediately for quenching and stored at −80°C until extraction. Extraction procedure for GC/MS and ion-pair LC/MS/MS was done as previously described (Putri et al., 2018).

For GC/MS analysis, extracted metabolites were derivatized by oximation and silylation prior to analysis. The oximation reagent, methoxyamine hydrochloride (Sigma-Aldrich, St. Louis, MO, United States) was first dissolved in pyridine (Wako, Osaka, Japan) to a concentration of 20 mg/mL and 50 μL was added to each sample tube containing the lyophilized extracts. After reaction at 30°C, 1200 rpm for 90 min, 25 μL of N-methyl-N-(trimethylsilyl)trifluoroacetamide (MSTFA) (GL Sciences, Tokyo, Japan) was added and the silylation reaction was performed at 37°C, 1200 rpm for 30 min. The derivatized samples were transferred to glass vials (Chromacol, Hertfordshire, United Kingdom) and analyzed within 24 h. For ion-pair LC/MS/MS analysis, extracted metabolites were dissolved in 100 μL of ultrapure water (Wako), vortexed, centrifuged at 16,000 × *g* for 3 min at 4°C and transferred to glass vials.

### Quantification of Extracellular Metabolites

Alcohols were quantified as previously described (Ohtake et al., 2017). Briefly, a GC-2010 system (Shimadzu) with a GL Science (Tokyo, Japan) InertCap Pure-WAX capillary column (30 m, 0.25 mm i.d., 0.25 μm film thickness) equipped with a flame ionization detector and an AOC-20i/s auto injector (Shimadzu) was used.

The remaining glucose in the culture was quantified using a F-kit D-glucose (Roche Diagnostics, Manheim, Germany) following the manufacturer’s instructions.

### GC/MS Analysis for Snapshot Metabolite Analysis

GC/MS analysis was performed as previous reported (Putri et al., 2018). Briefly, a GCMS-QP2010 Ultra (Shimadzu, Kyoto, Japan) gas chromatograph coupled with quadrupole mass spectrometer equipped with an AOC-20i/s auto injector (Shimadzu) was used with a CP-SIL 8 CB Low Bleed/MS column (30 m × 0.25 mm i.d., film thickness 0.25 mm, Agilent, Santa Clara, CA, United States). The raw data files were converted into netCDF (*.cdf) format according to the ANDI (Analytical Data Interchange Protocol) specification using the proprietary software GCMS solution (Shimadzu) before peak detection, baseline correction and retention time alignment using the freely available data processing tool MetAlign (Lommen, 2009). Data matrices from the alignment were then imported into AIoutput2 ver. 1.29 (Tsugawa et al., 2011) for automated Retention Index-based target compound identification and quantification.

### Ion-Pair LC/MS/MS Analysis for Snapshot Metabolite Analysis

Ion-pair LC/MS/MS analysis was performed as previous reported (Ohtake et al., 2017). Briefly, a Shimadzu Nexera UHPLC system coupled with LCMS 8030 Plus (Shimadzu) using a CERI L-column 2 ODS (150 mm × 2.1 mm, particle size 3 μm, Chemicals Evaluation and Research Institute, Tokyo, Japan) was used. The mobile phase (A) was 10 mM tributylamine and 15 mM acetate in water and mobile phase (B) was methanol. The raw data files were converted into *.abf file then imported into MRMPROBS (Tsugawa et al., 2013) to create data matrix.

### Multivariate Analysis

Principal component analysis (PCA) was performed using SIMCA-P+ version 14 (Umetrics, Umeå, Sweden). The metabolome data was normalized by an internal standard, mean centered and scaled to unit variance. *T*-test was performed using MS Excel to determine statistically significant differences.

## Results

### Systematic Identification of Metabolic Perturbations Caused by Overexpression of the Threonine Pathway

We initiated our study with comprehensive metabolic profiling to understand why overexpression of both the citramalate and threonine pathways did not have any effect on production titers. GC/MS and ion-pair LC/MS/MS analysis for snapshot metabolite analysis were performed on two previously constructed strains (Shen and Liao, 2013): SYN12 has expression of both the threonine and citramalate pathways, while SYN3 has expression of only the citramalate pathway and native expression of the threonine pathway. Each strain also contains an additional *leuA* knockout to prevent the downstream consumption of 2KB and conversion into 1-butanol or norvaline.

**FIGURE 2:**
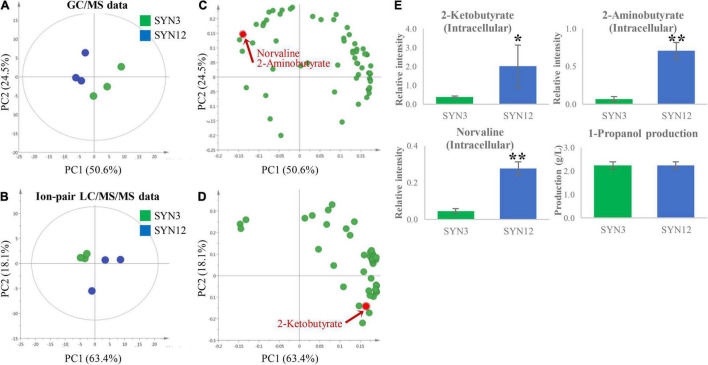
PCA analysis of SYN3 and SYN12. The score plot derived from autoscaled **(A)** GC/MS data. **(B)** Ion-pair LC/MS/MS data. The corresponding loading plot illustrating metabolites that contributed to the separation on PC1 and PC2 derived from autoscaled. **(C)** GC/MS data. **(D)** Ion-pair LC/MS/MS data. All metabolites contribution is shown in Supplementary Figure 1. **(E)** The result of intracellular key intermediate, by-products and 1-propanol production. Metabolite intensities shown in the *y*-axis were normalized to an internal standard. Asterisks indicate significant difference from the strain (*p < 0.05, **p < 0.01). The error bars indicate standard deviations obtained from three replicate fermentations.

Tentative identification of compounds was performed by comparison with our in-house library. Tentatively identified metabolites from GC/MS consisted of organic acids, sugars, amino acids, and other compounds, whereas the metabolites from ion-pair LC/MS/MS consisted of cofactors and central metabolites belonging to glycolysis, pentose phosphate pathway (PPP) and tricarboxylic acid cycle (TCA cycle) (Supplementary Table 2). The obtained data sets from GC/MS (63 annotated metabolites) and ion-pair LC/MS/MS analyses (41 annotated metabolites) were subsequently subjected to principal component analysis (PCA), an unsupervised multivariate analysis, to extract the most meaningful information from SYN3 and SYN12 strains. The resulting PCA score plot (Figures 2A–D) showed that PC1 separated samples based on different genotypes, while PC2 separated samples based on replicates. PC1 represented 50.6% (GC/MS) and 63.4% (ion-pair LC/MS/MS) of the total variance of the samples, respectively, while PC2 were 24.5% (GC/MS) and 18.1% (ion-pair LC/MS/MS), respectively. Then, compounds contributing to the separation of samples were examined in the loading plot.

Based on PCA analysis, several key pathway intermediates with significant perturbations between the two strains were revealed. Norvaline, 2-aminobutyrate, and 2KB were all accumulated at significantly higher concentrations within SYN12 (Figure 2E). Interestingly, the formation of norvaline is not expected, as the gene catalyzing the first step of the reaction from 2KB to 2-ketovalerate (2KV), *leuA*, has been deleted in both SYN3 and SYN12 strains. This initial finding led to the hypothesis that CimA, a key enzyme within the citramalate pathway and structural homolog to *leuA* (Atsumi and Liao, 2008), is catalyzing the conversion of 2KB to 2KV, and ultimately to norvaline and 1-butanol. Although the presence of CimA is likely causing this downstream conversion, the buildup of these side products nonetheless suggests that higher titers may be reached by further channeling metabolic flux toward 1-propanol production.

### Channeling Pathway Flux Highlights Pathway Bottlenecks

**FIGURE 3:**
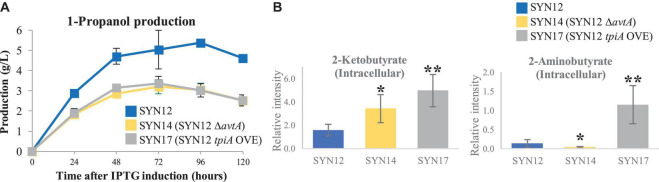
Effect of *avtA* deletion or *tpiA* overexpression. **(A)** 1-Propanol production. **(B)** Intracellular 2-ketobutyrate and 2-aminobutyrate. Samples were taken after 24 h of fermentation. Metabolite intensities shown in the *y*-axis were normalized to an internal standard. Asterisks indicate significant difference from the strain (*p < 0.05, **p < 0.01). The error bars indicate standard deviations obtained from three replicate fermentations.

Our initial attempt to improve production titers focused on preventing the formation of the unwanted metabolite 2-aminobutyrate. From SYN12, we knocked out *avtA* (to yield SYN14) to redirect flux toward 1-propanol production. However, a decrease in 1-propanol production was observed (Figure 3A). As expected, we observed a decrease in 2-aminobutyrate and an increase in 2KB compared to that of SYN12 (Figure 3B). Because 2KB is directly upstream of 1-propanol, the decrease in 1-propanol titer is counterintuitive, and may be a result of 2KB toxicity (Zhang et al., 2016; Fang et al., 2021).

A similar trend was observed in a separate experiment to supply carbon into the 1-propanol pathway. We examined the central metabolism and performed time course sampling in which we took samples every hour from 0 to 7 h after IPTG induction, at which point the cells were already growing in log phase. We performed ion-pair LC/MS/MS-based targeted analysis using 24 annotated metabolites belonging to glycolysis, TCA cycle, PPP, as well as important cofactors as listed in Supplementary Table 3. We found that dihydroxyacetone phosphate (DHAP) levels significantly increased over time (Supplementary Figure 2). To improve flux through glycolysis, we overexpressed *tpiA*, encoding triose-phosphate isomerase, which catalyzes the reversible reaction to convert DHAP to D-glyceraldehyde 3-phosphate (GAP) (Lee et al., 2013). Overexpression of *tpiA* in SYN12 (herein referred to as SYN17) resulted in approximately 3-fold increase of 2KB compared to the parental strain, accompanied by over 10-fold increase of downstream by-products derived from 2KB, 2-aminobutyrate (Figure 3B). Similar to SYN14 (SYN12 Δ*avtA*), a highly accumulated 2KB in the cell was severely detrimental for 1-propanol production (Figure 3A).

### Optimization of Alcohol Dehydrogenase Activity for Improved 1-Propanol Titers

Taken together, these results suggest that a bottleneck in 1-propanol biosynthesis may exist in either KivD or YqhD, those that convert 2KB into 1-propanol. We next attempted to improve titers by optimizing the activity of KivD and YqhD. We thus constructed two RBS libraries to vary the expression of KivD or YqhD by altering the RBS translation initiation rate. The Salis RBS Calculator v1.1 (Salis et al., 2009; Espah Borujeni et al., 2014) was used to design 8 variants per library using pAFC66 as a starting template (Table 1). The resulting library was cloned into SYN12 and a minimum of 23 colonies were screened for 1-propanol production after 24 h (Supplementary Figures 3A, 4), which ensured that all variants have been screened at least once with a 95% probability based on the Clarke Carbon formula.

**FIGURE 4:**
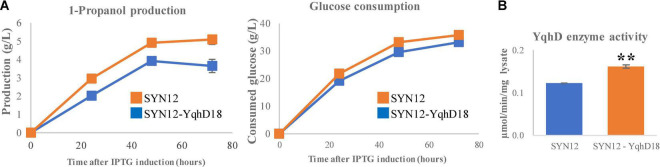
Effect of YqhD expression optimization. **(A)** 1-Propanol production and consumed glucose. **(B)** The relative specific activity of YqhD within cell lysate with improved RBS sequence. Asterisks indicate significant difference from the strain (**p < 0.01). The error bars indicate standard deviations obtained from three replicate fermentations.

From the screening, one library variant was identified from each that had at least a 30% increased titer compared to SYN12: these were denoted as SYN12-YdhD18 and SYN12-KivD34. We next assessed whether these strains exhibit changes in 1-propanol production. While SYN12-KivD34 did not show increased titers (Supplementary Figure 3B), SYN12-YqhD18 reached titers of 5.1 (g/L) and yield of 0.142 (g/g of glucose), compared to SYN12 that reached titers of 3.7 (g/L) and yield of 0.110 (g/g of glucose) in 72 h (Figure 4A). To verify that the altered RBS conferred an increase in YqhD activity, we measured the specific activity of YqhD *in vitro* in cell lysate and found a 33% increase in activity (Figure 4B).

## Discussion

The synergistic combination of both the citramalate pathway and threonine pathway to produce 1-propanol has been used previously to demonstrate improved theoretical yields and productivity. However, no change in titer has been observed with the overexpression of the threonine pathway over native levels. Here, we applied metabolomics-based strategies to understand the limitations of the synergistic pathway and to engineer SYN12 for increased 1-propanol titers.

The metabolomics strategies that were applied in this work contributed to a streamlined and logical series of strain improvement steps that ultimately allowed for increased production titers. Initial metabolomics analysis compared SYN3 and SYN12 to reveal an increase in concentrations of 2-aminobutyrate, norvaline, and 2-ketobutyrate in SYN12. SYN12 is additionally modified to overexpress the native threonine pathway. While no increase in titer was observed, the physiological consequences of the pathway overexpression became apparent in the accumulation of these upstream metabolites and byproducts.

We therefore sought to focus efforts on channeling the metabolic flux toward 1-propanol production. However, gene deletions of *avtA*, aimed at decreasing unwanted byproducts 2-aminobutyrate, had adverse effects on the production titers. Additional experiments aimed at increasing flux through glycolysis by overexpression of *tpiA*, and subsequently through the 1-propanol pathway, also resulted in a 2KB accumulation with a significant decrease in 1-propanol titers. 2KB is a short-chain alpha keto acid and a structural analog of pyruvate. As an indispensable metabolic intermediate in biological metabolism, 2-KB is found in the biosynthesis of L-isoleucine (Park et al., 2012; Yin et al., 2012), 2-aminobutyrate (Xu et al., 2019), 1-butanol (Atsumi and Liao, 2008), L-homoalanine (Zhang et al., 2010), and so on. However, because of its structural similarity with pyruvate, 2KB competitively inhibits enzymes used in pyruvate metabolism at high concentrations, which causes cellular toxicity (Zhang et al., 2016; Fang et al., 2021). As such, this suggests that enhancing the turnover rate of 2-KB to 1-propanol with KivD and YqhD may relieve toxicity caused by 2KB accumulation.

We then focused on optimizing flux of KivD and YqhD to relinquish this bottleneck. We thus constructed RBS libraries to vary the expression of each enzyme and isolated a variant. While SYN12-KivD34 increased KivD34 specific activity by 69%, it did not show increased titers (Supplementary Figures 3B,C). This might be due to the toxic intermediate propyl aldehyde, which can generate membrane lipid peroxidation (Pérez et al., 2008), thus suggesting that the turnover rate of propyl aldehyde to 1-propanol catalyzed by YqhD was not sufficient. SYN12-YqhD18, with a 33% increase in YqhD specific activity, exhibited a 38% increased titer and a 29% increased yield in 72 h compared to the base strain SYN12, which was the best 1-propanol producing strain constructed in Shen and Liao (2013). These results may imply that there was an insufficient turnover rate of toxic intermediates 2KB and propyl aldehyde into 1-propanol in SYN12. This highlights two potential future directions for further strain improvement. First, since propyl aldehyde is a toxic intermediate, it may be beneficial to simultaneously fine-tune expression of both of these pathway enzymes. However, generating RBS libraries to optimize both enzymes would result in a combinatorial library with a much greater number of variants, and thus, would require screening methods with larger throughput. Second, since turnover and toxicity of 2KB is improved with YqhD18, a future target for further engineering may be to increase flux through the pathway, possibly with overexpression of *tpiA* as demonstrated in SYN17. These future possibilities further highlight the value of metabolomics in the design-test-build cycle that is endemic to metabolic engineering and strain improvement.

Previous works have aimed at improving 1-propanol production in *E. coli* with various strategies. In one example, *E. coli* was modified to contain a keto-acid pathway to convert 2-ketobutyrate to propionyl-phosphate, which is then sequentially converted to propionate, propionyl-CoA, propionyl-aldehyde, and finally to 1-propanol (Choi et al., 2012). A separate study extended succinate dissimilation under anaerobic conditions (Srirangan et al., 2014). The titer reported here is much higher compared to other hosts such as in *Corynebacterium glutamicum* [12 mM (721 mg/L) from glucose] (Siebert and Wendisch, 2015), *Propionibacterium freudenreichii* (0.51 g/L from glycerol) (Ammar et al., 2013), and *Saccharomyces cerevisiae* (180 mg/L from glucose) (Nishimura et al., 2018). The efficient genetic tractability of *E. coli* allows for more complex genetic modifications than other species, and therefore *E. col*i has potential to be further engineered for higher 1-propanol production.

## Conclusion

The successful strain improvement in this work demonstrates the application of metabolomics-guided metabolic engineering strategies. More importantly, the data generated from metabolomics highlights metabolic perturbations that occur from pathway bottlenecks that may be relevant to the improvement of other production pathways.

## Data Availability Statement

The original contributions presented in the study are included in the article/Supplementary Material, further inquiries can be directed to the corresponding author/s.

## Author Contributions

KN, NK, and WL: metabolite measurement and data curation and analysis. TO: data curation and interpretation, and writing the manuscript. SPu: metabolite measurement, data analysis and interpretation, supervision, and writing the manuscript. CS: strain construction, data analysis and interpretation, and revising manuscript. SPo: strain construction, data analysis and interpretation, and writing the manuscript. EF and JL: supervision, project management, and resources. All authors have read and approved the final manuscript.

## Conflict of Interest

The authors declare that the research was conducted in the absence of any commercial or financial relationships that could be construed as a potential conflict of interest.

## Publisher’s Note

All claims expressed in this article are solely those of the authors and do not necessarily represent those of their affiliated organizations, or those of the publisher, the editors and the reviewers. Any product that may be evaluated in this article, or claim that may be made by its manufacturer, is not guaranteed or endorsed by the publisher.
