# Supplementary material for: Metabolomics-Driven Identification of the Rate-Limiting Steps in 1-Propanol Production
Source: Front Microbiol. 2022 Apr 14;13:871624. doi: 10.3389/fmicb.2022.871624 (PMC9048197; doi:10.3389/fmicb.2022.871624)

**Supplementary Table 1** Primers used in this work.

| **Name** | **Relevant characteristics** | **Source** |
| --- | --- | --- |
| **Primer** |  | Plasmid |
| CS92 tpiA f asm | tgatgaatcatcagtaaaaggagatataccATGCGACATCCTTTAGTGA  TGGG | pCS92 |
| CS93 tpiA r asm | TCGTTTTATTTGATGCCTCTAGCACGCGTATTAAGCCT  GTTTAGCCGCTTCTG | pCS93 |
| CS94 pCS49 r asm | AGGATGTCGCATGGTATATCTCCTTTTACTGATGATTC  ATCATCAATTTACGCA | pCS94 |
| CS95 pCS49 f asm | tgcagaagcggctaaacaggcttaaTACGCGTGCTAGAGGCATCAAA  TA | pCS95 |
| CS98 Plac f asm | Ttgtgagcggataacaagatactgagcacatcagcaggac | pCS98 |
| CS99 Plac r asm | GTCCTGCTGATGTGCTCAGTATCTTGTTATCCGCTCAC  AA | pCS99 |
|  |  |  |
| KivD lib F | GTAATCTCCTACTGTATACATGAAKACCTCCYTATCTTT  GASTATTTTATTGATGGTCAGTGCGTCCTGCTGA | This study |
| KivD lib R | TAATCTCCTACTGTATACATGTTGATACCTCSTTATTAT  ATTATTMATAGGTSGTCAGTGCGTCCTGCTG | This study |
| YqhD lib 1F | CTATTTAATAKCCTGGAGKTACTTAATGAACAACTTTAA  TCTGCACACCC | This study |
| YqhD lib 1R | AMCTCCAGGMTATTAAATAGTTATGATTTATTTTGTTC  AGCAAATAGTTTG | This study |
| YqhD lib 2F | CCCGGCTGGCTATAGAGVGGTATATAATGAACAACTTT  AATCTGCACACCC | This study |
| YqhD lib 2R | ACCBCTCTATAGCCAGCCGGGTTATGATTTATTTTGTT  CAGCAAATAGTTTG | This study |
| YqhD lib 3F | TCCTCCTTTCTGACTCGCGAGGTGCGGCTTATGATTTA  TTTTGTTCAGCAAATAGTTT | This study |
| YqhD lib 3R | TCGCGAGTCAGAAAGGAGGAACCCGCATGAACAACTTT  AATCTGCACACCC | This study |

**Supplementary Table 2** A list of tentatively identified metabolites from GC/MS and ion-pair LC/MS/MS analyses.

| No | Compound Name | |
| --- | --- | --- |
|  | GC/MS | Ion-pair LC/MS/MS |
| 1 | 2-Aminobutyric acid | 1,3-BPG |
| 2 | 2-Aminoethanol | 2-Amino adipic acid |
| 3 | 2-HydroxyButyrate | 2-ketobutyrate |
| 4 | 2-Hydroxypyridine | 2-Oxoglutarate |
| 5 | 3-Hydroxybutyrate | 3PGA |
| 6 | 3-Methylglutarate | 6-Phosphogluconate |
| 7 | 4-Aminobutyric acid | Acetyl CoA |
| 8 | 4-Hydroxyphenethyl alcohol (Tyrosol) | ADP |
| 9 | Adenine | a-Glycerophosphate |
| 10 | Alanine | AMP |
| 11 | Aspartic acid | ATP |
| 12 | Cadaverine | CDP |
| 13 | Citramalic acid | CMP |
| 14 | Citric acid + Isocitric acid | CTP |
| 15 | Fructose | Cytidine |
| 16 | Fumaric acid | DHAP |
| 17 | Galactose | F1,6P |
| 18 | Glutamic acid | F2,6P |
| 19 | Glutamine | FAD |
| 20 | Glutaric acid | GDP |
| 21 | Glyceric acid | GMP |
| 22 | Glycerol | GTP |
| 23 | Glycine | Guanine |
| 24 | Glycolic acid | Guanosine |
| 25 | homoserine | Inosine |
| 26 | Hypoxanthine | Lactate |
| 27 | Isoleucine | NAD |
| 28 | Lactic acid | NADH |
| 29 | Lactitol | NADP |
| 30 | Leucine | Orotate |
| 31 | Lyxose | Pantothenate |
| 32 | Maleic acid | Phophoenolpyruvate |
| 33 | Malic acid | Pyruvate |
| 34 | Melibiose | S7P |
| 35 | meso-erythritol | Thymine |
| 36 | n-Butylamine | TMP |
| 37 | N-Carbamoyl-L-Aspartate | UDP |
| 38 | Nicotinic acid | UDP-Glu |
| 39 | Norvaline | UMP |
| 40 | n-Propylamine | Uridine |
| 41 | Octadecanoate | UTP |
| 42 | Ornithine |  |
| 43 | Orotic acid |  |
| 44 | Oxalate |  |
| 45 | Phenylalanine |  |
| 46 | Phosphate |  |
| 47 | Proline |  |
| 48 | Psicose (or Tagatose) |  |
| 49 | Putrescine |  |
| 50 | Pyroglutamic acid |  |
| 51 | Pyruvate+Oxalacetic acid |  |
| 52 | Serine |  |
| 53 | Sorbitol |  |
| 54 | Succinic acid (or aldehyde) |  |
| 55 | Threonine |  |
| 56 | Thymine |  |
| 57 | Trehalose |  |
| 58 | Tryptophan |  |
| 59 | Tyrosine |  |
| 60 | Uracil |  |
| 61 | Urea |  |
| 62 | Valine |  |
| 63 | Xylulose |  |

**Supplementary Table 3** A list of metabolites from targeted ion-pair LC/MS/MS analysis.

| Central metabolism | Nucleotides | Cofactors | Others |
| --- | --- | --- | --- |
| 1,3-BPG | ATP | NADH | 2-Oxoglutarate |
| 3PGA+2PGA | ADP | NADP | Acetyl-CoA |
| 6-Phosphogluconate |  | NAD | Citrate |
| DHAP |  |  | Fumalate |
| F6P |  |  | Isocitrate |
| FBP |  |  | Malate |
| Phophoenolpyrvate |  |  | Succinate |
| Pyruvate |  |  |  |
| R5P |  |  |  |
| Ru5P |  |  |  |
| S7P |  |  |  |

**Supplementary Figure 1** All metabolites contribution in loading plot of PCA in Figure 2. (A) All metabolites contribution in Figure 2C. (B) All metabolites contribution in Figure 2D.


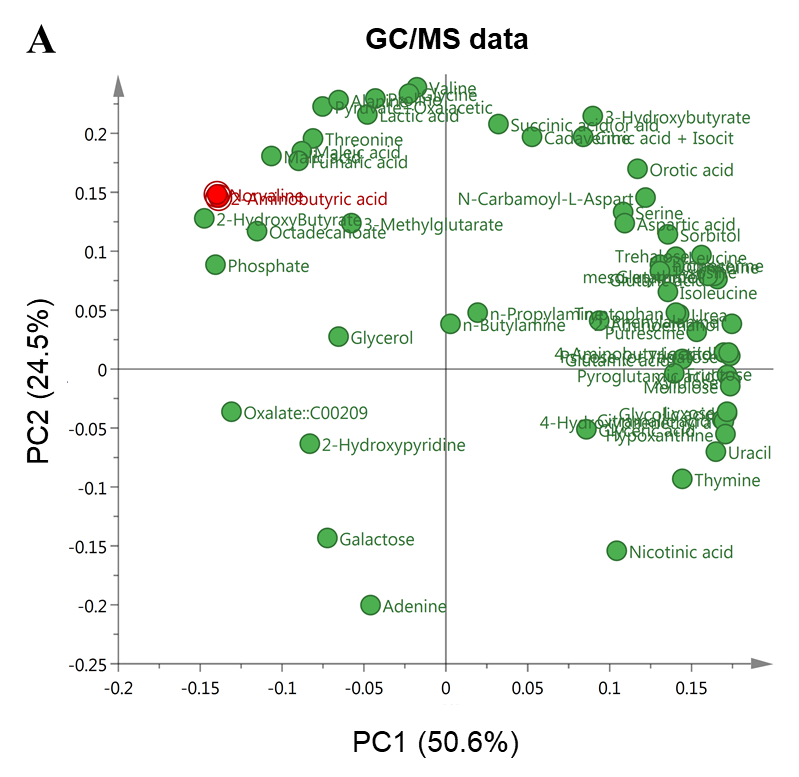


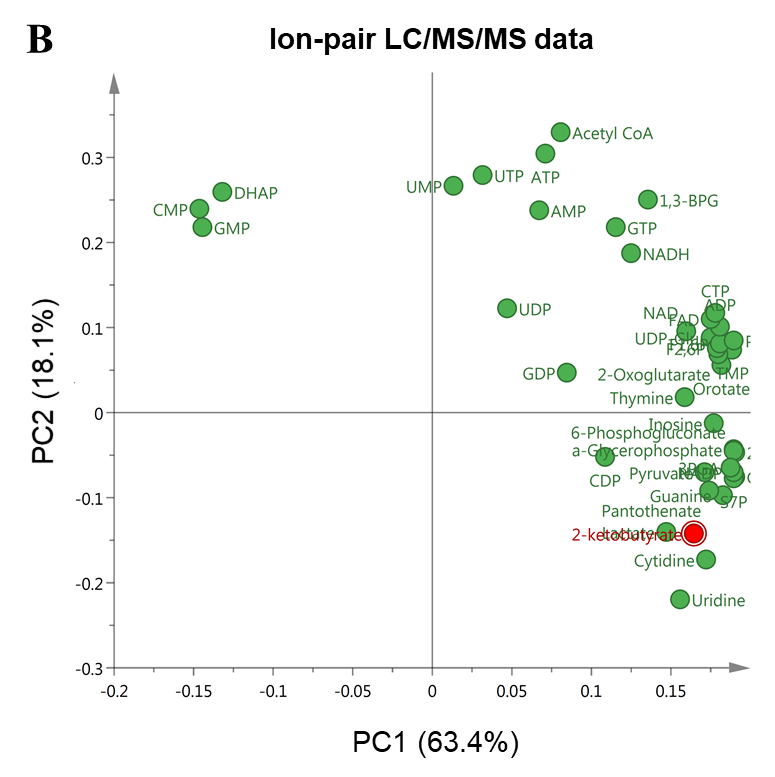


**Supplementary Figure 2** Time course targeted analysis showing intracellular DHAP. Metabolite intensities shown in the y-axis were normalized to an internal standard. The error bars indicate standard deviations obtained from three replicate fermentations.


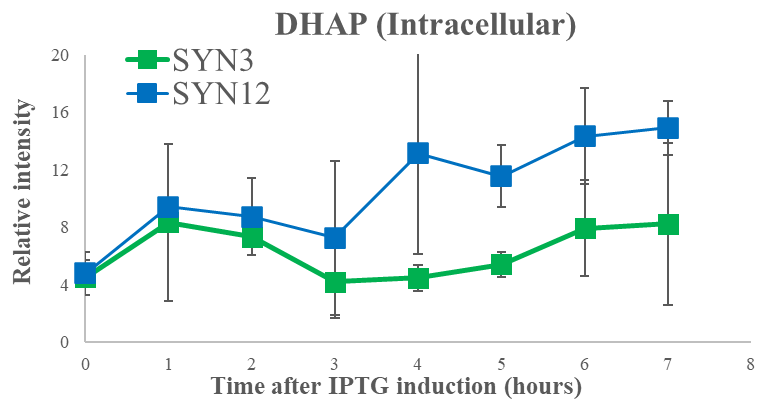


**Supplementary Figure 3** (A) 1-Propanol production titer of each colony harboring KivD variant. Samples were taken after 24 hours of fermentation in test tubes. Dashed line represents 2.5 g/L of 1-propanol production titer. (B) Additional experiment to confirm 1-propanol production titer in SYN12-KivD34. Samples were taken after 24 hours of fermentation. The error bars indicate standard deviations obtained from three replicate fermentations. (C) The relative specific activity of KivD within cell lysate with improved RBS sequence. Asterisks indicate significant difference from the strain (**: p < 0.01). The error bars indicate standard deviations obtained from three replicate fermentations.


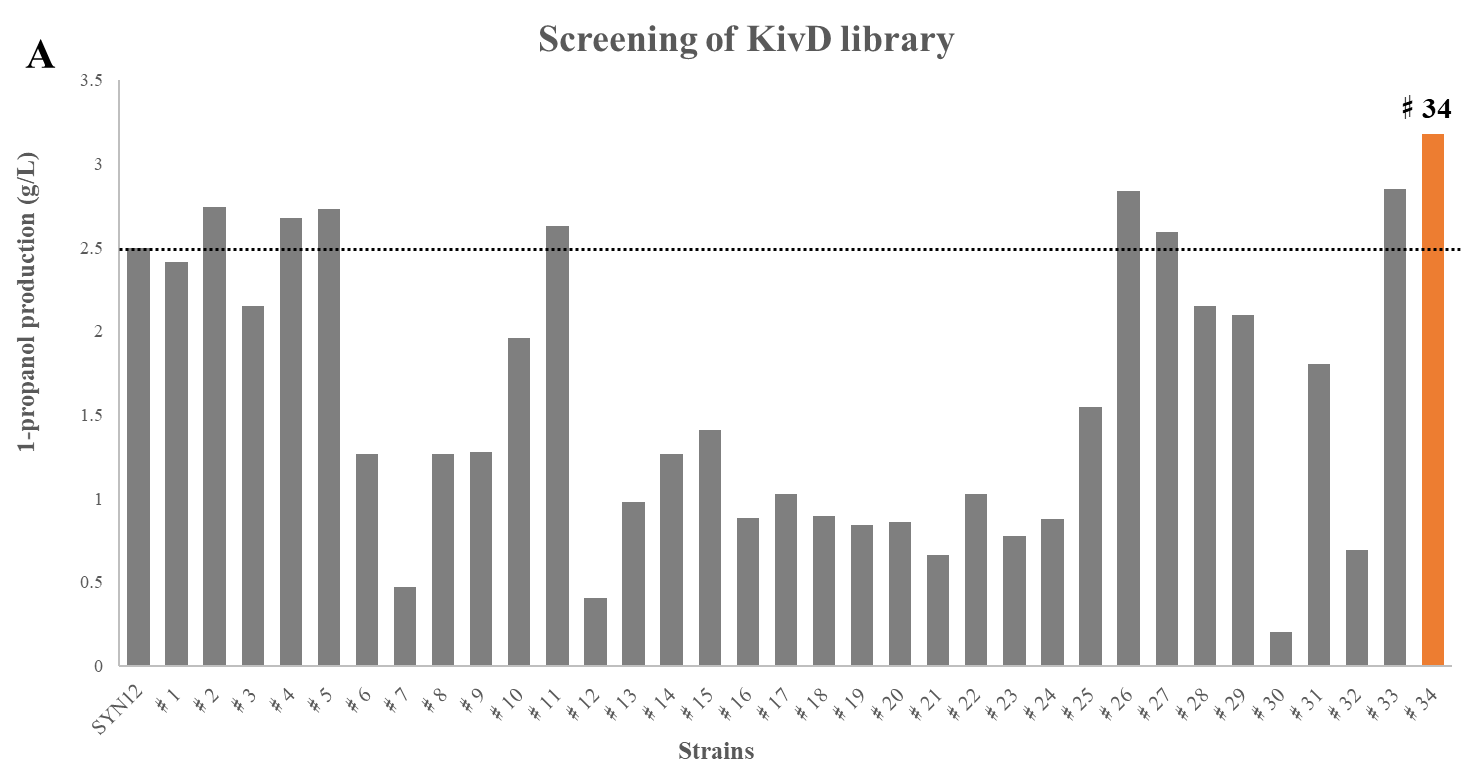


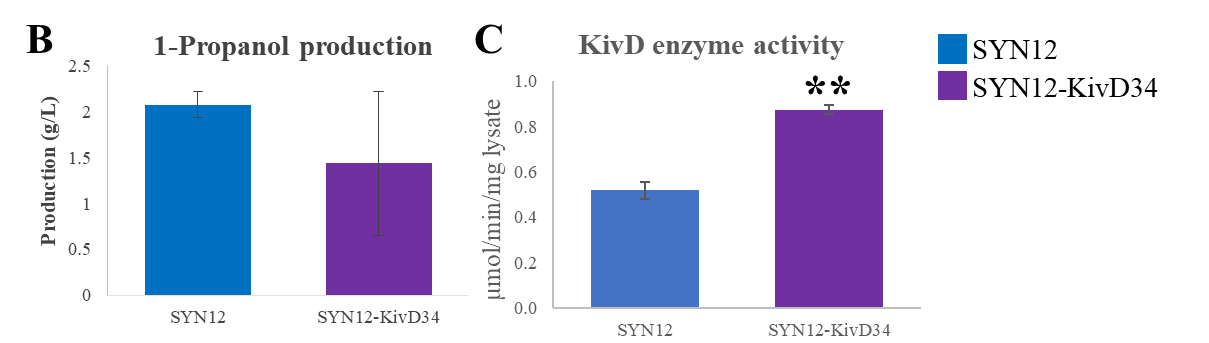


**Supplementary Figure 4** 1-Propanol production titer of each colony harboring YqhD variant. Samples were taken after 24 hours of fermentation in test tubes. Dashed line represents 2.7 g/L of 1-propanol production titer.


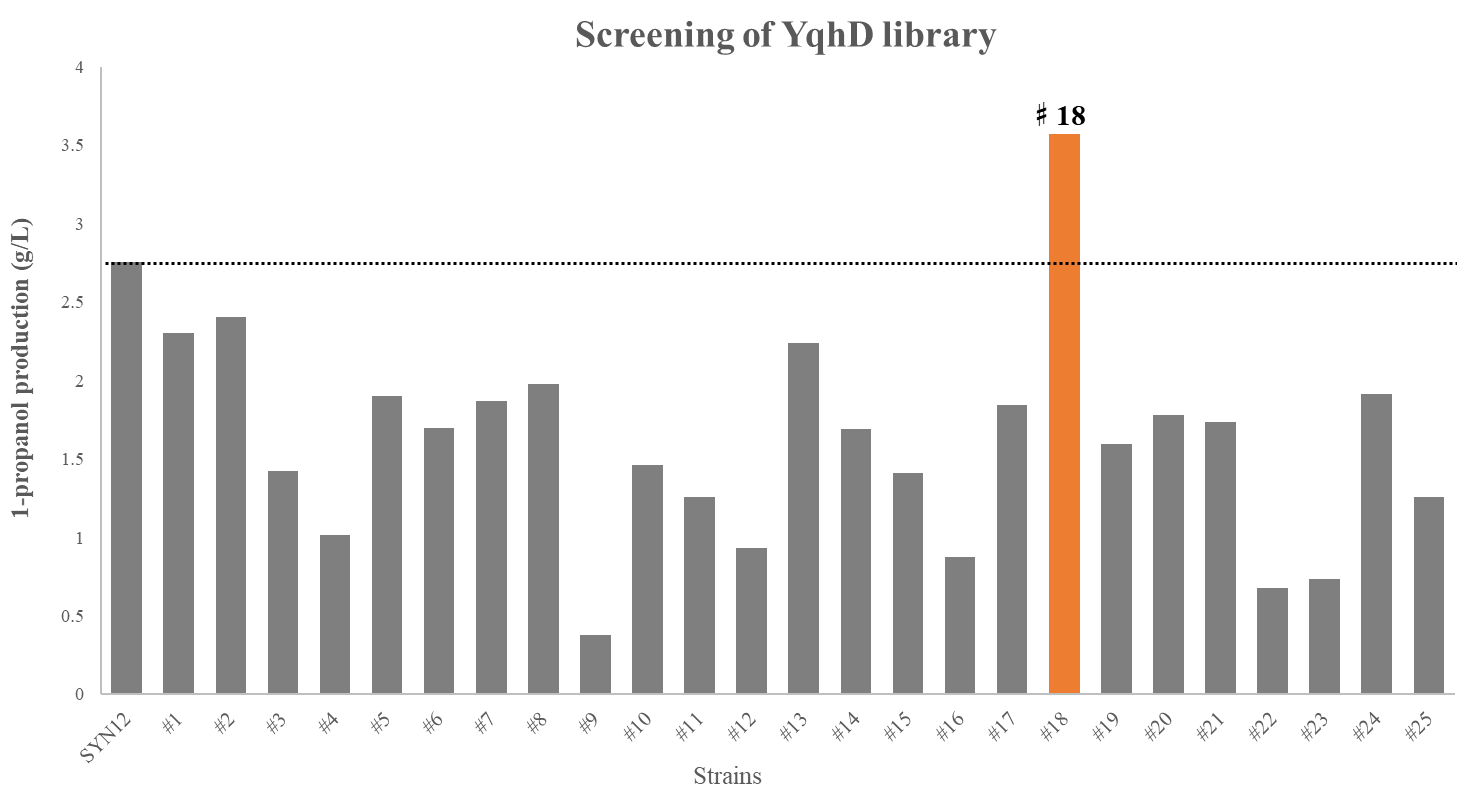

Supplement: Supplementary file 1 [file Data_Sheet_2.docx]
